# Trends in surgical approaches and adnexal surgeries during hysterectomies for benign diseases between 2015–2021

**DOI:** 10.3389/fsurg.2023.1068776

**Published:** 2023-02-20

**Authors:** Xuejiao Zhu, Hongli Xi, Zhifang Li, Xinping Wang

**Affiliations:** Department of Obstetrics and Gynecology, Xiangyang No.1 People’s Hospital, Hubei University of Medicine, Xiangyang, China

**Keywords:** gynaecologic surgical procedures, hysterectomy, salpingectomy, salpingo-oophorectomy, surgical

## Abstract

**Background:**

Hysterectomy is a widely used surgical approach for benign gynecological conditions, although recently there have been differences in the surgical route selected in different regions.

**Aims:**

To estimate recent temporal trends, this study collected data on surgical approaches and adnexal surgeries during hysterectomies for benign diseases at a single institute from 2015 to 2021.

**Materials and methods:**

We retrospectively analyzed data from Xiangyang No.1 People's Hospital, Hubei University of Medicine in Xiangyang, China, and identified 1828 women who underwent hysterectomies for benign gynecologic conditions performed with or without bilateral salpingectomy (BS) or bilateral salpingo-oophorectomy (BSO) between January 2015 and December 2021.

**Results:**

There was an upward trend in the performance of hysterectomy and hysterectomy with BS, and there was a difference in the trends of concomitant adnexal surgery between AH, TLH, and VH, especially in TLH with BS. Patient characteristics data demonstrated that the most frequent indication for hysterectomy was leiomyoma, especially in women aged 45 to 65. Compared to AH, TLH, and VH, the operative bleeding, duration of surgery, and length of hospital stays of patients undergoing TLH with BS and BSO was the lowest. The surgical approach to benign diseases has changed dramatically due to a growing proportion of patients choosing minimally invasive procedures. The laparoscopic approach is becoming popular due to its capacity to decrease intraoperative blood loss and reduce hospitalization.

**Conclusions:**

We should put more emphasis on surgical training for the TLH approach and help gynecologic surgeons provide the proposed added benefit of BS to their patients.

## Introduction

Despite the remarkable impact of conservative management of benign uterine disease and the decline in hysterectomy rates, hysterectomy remains the most common and widely performed gynecologic surgical procedure worldwide. Hysterectomy is the treatment of choice for 90% of benign uterine diseases (1). Options for hysterectomies include abdominal, vaginal, laparoscopic, and robotic procedures (2, 3). Common benign gynecological conditions treated by hysterectomy include uterine leiomyomata, endometriosis, abnormal menstrual bleeding, and pelvic organ prolapse, among others (4).

Bilateral salpingectomy (BS) is now widely studied, and two recent population-based retrospective studies have shown a decreased risk of ovarian cancer among women receiving salpingectomy or tubal ligation (5, 6). Bilateral salpingo-oophorectomy (BSO; the removal of both ovaries and fallopian tubes) is also commonly used in clinical hysterectomy, which is the most common procedure for benign uterine disease in nonpregnant women worldwide and is effective in preventing ovarian cancer and reducing the risk of subsequent surgery. However, BSO is not suitable for all women as it results in immediate menopause, which may lead to increased mortality due to cardiovascular disease, cognitive impairment, sexual dysfunction, and osteoporosis (7). Therefore, the technical trend of hysterectomy is determining the appropriate surgical approach to reduce the risk of hysterectomy itself.

Although surgical approaches have been regulated in numerous reports and published guidelines, there is still substantial variation within and between countries about the choice of surgical route (8–10). This study aims to examine changes in surgical approach and adnexal surgeries during hysterectomies for benign diseases from 2015 to 2021. This paper mainly focuses on the influence of abdominal, total laparoscopic, and vaginal surgical approaches on hysterectomies for benign indications.

## Patients and methods

A retrospective study was conducted to collect 1828 cases of benign hysterectomy from 01/01/2015 to 31/12/2021 from the electronic case database of the Department of Obstetrics and Gynecology of the No. 1 People's Hospital of Xiangyang City, Hubei University of Medicine (a large general hospital in Xiangyang City, Hubei Province, China). Case searches were performed using the following ICD-9-CM procedure codes: abdominal hysterectomy (AH) (68.3), total laparoscopic hysterectomy (TLH) (68.4), or vaginal hysterectomy (VH) (68.5). AH requires an incision of the abdominal wall, separation of the bladder, and treatment of blood vessels and ligaments before a hysterectomy. TLH used a four-hole method of abdominal distension, a puncture cannula, and an insertion scope to remove the uterus under laparoscopy. VH is the removal of the uterus through the vagina, with the incision located at the top of the vaginal vault, leaving no wound in the abdomen. This study was approved by the Ethics Committee of Xiangyang No. 1 People's Hospital, Hubei University of Medicine (2022KY058). In accordance with the Declaration of Helsinki, all patients provided written or oral informed consent prior to enrollment.

Case inclusion criteria included a clinical diagnosis of benign uterine diseases, the main surgical approaches of AH, TLH, or VH, the absence of surgical contraindications, normal cognition, and the completeness of clinical records. Excluded criteria included female patients with ICD-9-CM diagnosis codes for primary or secondary malignant diseases (140–208), complicated by important organ dysfunction, comorbid mental illness, severe respiratory diseases, missing clinical data, non-cooperation, or involuntary participation in this study. We subclassified subtotal AH, TLH, and VH according to the type of adjoint surgery performed at the time of hysterectomy, specifically bilateral salpingectomy (BS) and bilateral salpingo-oophorectomy (BSO). Clinical data such as operative bleeding, duration of surgery, length of hospital stays, surgery cost, and uterine volume were also collected.

Statistical analyses were performed using SPSS 22.0; *P* < 0.05 was considered statistically significant.

## Results

**Figure 1:**
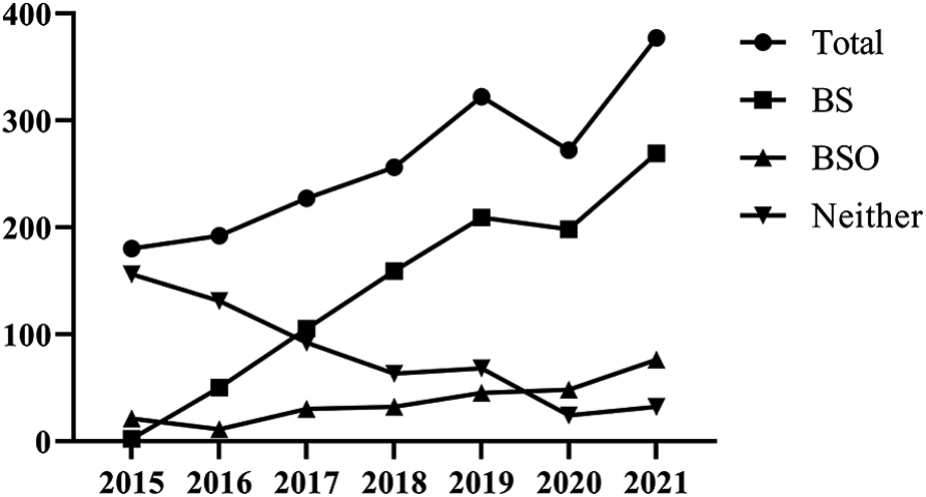
Total number of hysterectomies performed, BS, BSO, and neither.

Between January 2015 and December 2021, 1,828 women between the ages of 29 and 83 years had hysterectomies for benign gynecologic conditions. Figure 1 shows the total number of patients who underwent AH, TLH, or VH with BS, BSO, or neither BS nor BOS, in addition to hysterectomies with BS, BSO, and neither. During the study period, a regular increase in the total number of hysterectomies was observed between 2015 and 2019. Compared to 2019, there were −15.53% (*n* = 272) fewer hysterectomies in total in 2020. In 2021, a 38,60% increase (*n* = 377) was observed in comparison to 2020. The trend for the number of hysterectomies with BS mirrored that for the total number of hysterectomies. In addition, there was a steady increase in the number of hysterectomies with BSO. Moreover, neither BS nor BSO showed a declining trend during the research phase.

**Figure 2:**
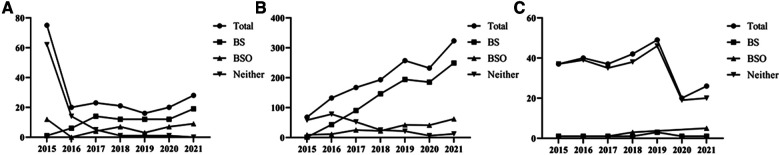
The total number of hysterectomies, the number of hysterectomies with BS, BSO, and neither at AH (**A**), TLH (**B**), and VH (**C**).

Figure 2 shows the total number of hysterectomies, the number of hysterectomies with BS, BSO, or neither in each surgical method. The average annual growth rate of the total number of hysterectomies was −17.89% in AH, 36.56% in TLH, and −6.81% in VH. In the AH (Figure 2A), there was a sharp downward trend of −73.33% between 2015 and 2016 in the total number of AH and the number of AH without BS and BSO. Accordingly, the total number of AHs stayed stable between 2016 and 2019 and presented a marginal increase between 2019 and 2021. However, the number of AH without BS and BSO decreased slightly in 2016–2018 and bottomed out in 2018–2021. The number of AH with BS presented a marginal upward trend in 2015–2017, then flattened out in 2017–2020, and finally grew in 2020–2021.

The number of AH with BSO remained low level throughout the study period. In the TLH (Figure 2B), the total number of TLH and the number of TLH with BS increased steadily between 2015 and 2019, declined by 9.73% and 4.64%, respectively, in 2021 in comparison to 2019, and exhibited an upward trend again in 2020–2021. Meanwhile, the number of TLH without BS and BSO began to rise and then dwindled, and the number of TLH with BSO presented a marginal upward trend during the study period. In the VH (Figure 2C), both the total number of VH and the number of VH without BS and BSO grew initially and then gradually diminished between 2015 and 2019, but 2019 was characterized by a decline followed by an increase in 2021. In contrast, the number of VH cases with BS and BSO remained low between 2015 and 2021.

Table 1 provides a breakdown of patient characteristics by indication for AH, TLH, and VH. Leiomyoma was the most frequent indication for AH, TLH, and VH in relation to gestational ages, followed by endometrial lesions. In the age groups 25–40 and 40–45, endometrial lesions were the most common indication for AH, TLH, and VH (*n* = 30, 69.7%, and *n* = 115, 52.5%), followed by leiomyomas (*n* = 8, 18.6%, and *n* = 77, 35.2%). In the age groups 45–50 and 50–65, most of the indications for AH, TLH, and VH were leiomyomas (*n* = 336, 51.4%, and *n* = 344, 47.1%), followed by endometrial lesions (*n* = 263, 40.3%, and *n* = 141, 19.3%). In the age group >65, the majority of indications for AH, TLH, and VH were uterine prolapse (*n* = 130, 71.0%), followed by leiomyoma (*n* = 15, 8.2%), other diseases (*n* = 24, 13.1%), and cervical intraepithelial neoplasia (*n* = 14, 7.7%).

**Table 1:** Patient characteristics by hysterectomy indication.

Variable	Category	Indication for hysterectomy, *n* (%)				
		Leiomyoma	Endometrial lesions	Uterine prolapse	Cervical intraepithelial neoplasia	Other diseases
Gravidity	0	5 (41.7%)	2 (16.7%)	1 (8.3%)	1 (8.3%)	3 (25.0%)
	1	79 (47.0%)	54 (32.1%)	15 (8.9%)	11 (6.6%)	9 (5.4%)
	2	235 (52.2%)	115 (25.6%)	47 (10.4%)	34 (7.6%)	19 (4.2%)
	3	231 (43.4%)	170 (32.0%)	74 (13.9%)	32 (6.0%)	25 (4.7%)
	>3	229 (34.4%)	207 (31.1%)	128 (19.2%)	65 (9.7%)	37 (5.6%)
Age group, year	25–40	8 (18.6%)	30 (69.7%)	2 (4.7%)	2 (4.7%)	1 (2.3%)
	40–45	77 (35.2%)	115 (52.5%)	2 (0.9%)	23 (10.5%)	2 (0.9%)
	45–50	336 (51.4%)	263 (40.3%)	9 (1.4%)	35 (5.4%)	10 (1.5%)
	50–65	344 (47.1%)	141 (19.3%)	122 (16.7%)	69 (9.5%)	54 (7.4%)
	>65	15 (8.2%)	0 (0.0%)	130 (71.0%)	14 (7.7%)	24 (13.1%)

Note: Weighted to estimate national frequency; the sum of all groups may not add up to the total due to missing data.

Details of patients' characteristics by surgical approach are given in Table 2. Patients undergoing hysterectomies have increased number of pregnancies. In terms of AH, the majority of participants were aged 50–65 (*n* = 86, 42.3%), followed by those aged 45–50 (*n* = 83, 40.9%), 40–45 (*n* = 24, 11.8%), >65 (*n* = 6, 3.0%), and 25–40 (*n* = 4, 2.0%). The majority of TLH participants were aged 45–50 (*n* = 562, 40.9%), followed by those aged 50–65 (*n* = 528, 38.4%), 40–45 (*n* = 192, 14.0%), >65 (*n* = 55, 4.0%), and 25–40 (*n* = 37, 2.7%). The majority of age groups in the VH were >65 (*n* = 122, 48.6%), followed by 50–65 (*n* = 117, 46.6%), 45–50 (*n* = 7, 2.8%), 40–45 (*n* = 3, 1.2%), and 25–40 (*n* = 2, 0.8%). In the AH, leiomyoma (*n* = 117, 57.6%) was the most frequent indication for hysterectomy, followed by endometrial lesions (*n* = 47, 23.2%), other diseases (*n* = 31, 15.3%), cervical intraepithelial neoplasia (*n* = 7, 3.4%), and uterine prolapse (*n* = 1, 0.5%). In the TLH, leiomyoma was the most frequent reason for hysterectomy (*n* = 659, 48.0%), followed by endometrial lesions (*n* = 498, 36.2%), cervical intraepithelial neoplasia (*n* = 132, 9.6%), other diseases (*n* = 56, 4.1%), and uterine prolapse (*n* = 29, 2.1%). In the VH, the most frequent indication for hysterectomy was uterine prolapse (*n* = 235, 93.6%), followed by endometrial lesions, cervical intraepithelial neoplasia, other diseases, and uterine prolapse (both *n* = 4, 1.6%).

**Table 2:** Patient characteristics by surgical approach.

Variable	Category	Surgical approach for hysterectomy, *n* (%)		
		AH	TLH	VH
Gravidity	0	2 (1.0%)	9 (0.7%)	1 (0.4%)
	1	19 (9.4%)	139 (10.1%)	13 (5.2%)
	2	58 (28.6%)	349 (25.4%)	42 (16.7%)
	3	60 (29.5%)	396 (28.8%)	73 (29.1%)
	>3	64 (31.5%)	481 (35.0%)	122 (48.6%)
Age group, year	25–40	4 (2.0%)	37 (2.7%)	2 (0.8%)
	40–45	24 (11.8%)	192 (14.0%)	3 (1.2%)
	45–50	83 (40.9%)	562 (40.9%)	7 (2.8%)
	50–65	86 (42.3%)	528 (38.4%)	117 (46.6%)
	>65	6 (3.0%)	55 (4.0%)	122 (48.6%)
Indication for hysterectomy	Leiomyoma	117 (57.6%)	659 (48.0%)	4 (1.6%)
	Endometrial lesions	47 (23.2%)	498 (36.2%)	4 (1.6%)
	uterine prolapse	1 (0.5%)	29 (2.1%)	235 (93.6%)
	Cervical intraepithelial neoplasia	7 (3.4%)	132 (9.6%)	4 (1.6%)
	Other diseases	31 (15.3%)	56 (4.1%)	4 (1.6%)

Note: TLH, total laparoscopic hysterectomy. Weighted to estimate national frequency; the sum of all groups may not add up to the total due to missing data.

Table 3 divides patients undergoing hysterectomy with BS or BSO by different surgical approaches to hysterectomy for benign indications and records hospitalization information for patients, including operative bleeding, duration of surgery, length of hospital stays, surgery cost, and uterine volume. During the five-year study period, a total of 1,828 women were included in the study, with 203 undergoing AH, 1,374 undergoing TLH, and 251 undergoing VH. Specifically, 76 patients underwent a BS (37.4%), 42 BSO (20.7%), and 85 neither (41.9%) at AH, 909 patients underwent a BS (66.2%), 212 BSO (15.4%), and 253 neither (18.4%) at TLH, and eight patients underwent a BS (3.2%), 9 BSO (3.6%), and 234 neither (93.2%) at VH. Furthermore, there were notable differences in the severity of operative bleeding for those patients undergoing AH, TLH, and VH with BS (*P* < 0.05), with VH having the highest incidence. The operative bleeding of patients undergoing VH was significantly higher than TLH (*P* < 0.05) and VH was significantly higher than AH (*P* < 0.05). There were also notable differences in the operative bleeding of patients undergoing AH, TLH, and VH with BSO (*P* < 0.05), with AH having the highest operative bleeding and being significantly higher than TLH (*P* < 0.05). Patients undergoing hysterectomy without BS and BSO were found to have statistically different operative bleeding among AH, TLH, and VH (*P* < 0.05), with AH having the highest operative bleeding. The operative time of patients undergoing AH, TLH, and VH with BS and BSO (*P* < 0.05) differed significantly, with TLH requiring more time than VH. In terms of length of stay, there was a statistically significant difference between BS patients and neither BS nor BSO patients among the three surgical approaches. The greatest operative bleeding was AH among three surgical approaches for patients with BSO and the greatest operative bleeding was VH among three surgical approaches for patients without BS nor BSO. In terms of surgery cost, there were significant differences between the three surgical approaches with BS, BSO, and neither BS nor BSO (*P* < 0.05) with VH having the highest surgery cost among the three surgical approaches with BS, BSO, and neither BS nor BSO. In terms of uterine volume, among the three surgical methods, the differences between BS patients, BSO patients and patients without BS or BSO were statistically significant. Among them, the three types of VH surgical approach had the largest uterine volume.

**Table 3:** Comparison of rates of bilateral salpingectomy (BS) and bilateral salpingo-oophorectomy (BSO) between different surgical approaches to hysterectomy for benign indications.

Variable	AH (*n* = 203)			TLH (*n* = 1374)			VH (*n* = 251)		
	BS	BSO	Neither	BS	BSO	Neither	BS	BSO	Neither
Total	76 (37.4%)	42 (20.7%)	85 (41.9%)	909 (66.2%)	212 (15.4%)	253 (18.4%)	8 (3.2%)	9 (3.6%)	234 (93.2%)
Operative bleeding, ml	222.89 ± 157.74^a^	163.33 ± 146.61^a^	295.59 ± 338.21^a^	168.19 ± 197.23^b^	114.55 ± 104.10^b^	204.88 ± 168.73^b^	343.75 ± 219.48^a,c^	144.44 ± 84.57	145.14 ± 89.15^c^
Duration of surgery, min	135.53 ± 36.34^a^	133.21 ± 46.10^a^	132.50 ± 36.77	132.86 ± 47.83^a,c^	126.74 ± 49.74^a^	134.68 ± 50.96	188.75 ± 64.85^b^	165.89 ± 40.89^b^	126.02 ± 36.00
Length of hospital stay, d	11.57 ± 6.17	12.74 ± 3.95^a^	12.53 ± 2.86^a^	10.18 ± 3.33	11.00 ± 3.11^b^	11.31 ± 3.36^b^	13.00 ± 7.27	12.44 ± 4.64	13.27 ± 3.52^c^
Surgery cost, ¥	15,386.02 ± 4338.07^a^	15,879.04 ± 4988.37^a^	11,305.74 ± 4117.25^a^	17,505.86 ± 3396.33^b^	17,099.19 ± 3579.64^ac^	15,286.29 ± 3320.87^b^	17,585.02 ± 3875.19	20,936.33 ± 5610.66^b^	11,408.53 ± 3193.65^a,c^
Uterine volume, cm^3^	1763.99 ± 3050.59^a^	892.67 ± 1329.98	802.36 ± 1200.94	580.92 ± 387.43^b^	258.95 ± 330.77	498.69 ± 299.90	353.75 ± 242.93^c^	115.33 ± 110.99	53.15 ± 76.52

Note: BS, bilateral salpingectomy; BSO, bilateral salpingo-oophorectomy. Different letters indicate a significant difference among groups of surgical approaches (*P* < 0.05). Weighted to estimate national frequency; the sum of all groups may not be added up to the total due to missing data.

^a^^,c^Represent the data less than the control group with significant difference.

^b^Represents the data greater than the control group with significant difference.

## Discussion

During the study period, our hospital observed an important shift in the surgical approaches and adnexal procedures performed during hysterectomies for benign gynecologic conditions. The total number of patients undergoing AH, TLH, and VH and the number of patients undergoing AH, TLH, and VH with BS in this study have increased steadily since 2015, whereas the number of patients undergoing AH, TLH, and VH with BSO has increased only slightly. A similar study found that gynecologic surgeons in the United States were increasingly performing BS during benign hysterectomy (11), and that nearly 60% of women had undergone opportunistic salpingectomy during benign hysterectomy by 2015 (11). In addition, our study revealed a limitation of BSO during VH, similar to a recent study that found that 16.5% of VH were performed with salpingectomy compared to 55.8% of hysterectomies performed *via* other routes (12). Despite the fact that performing adnexal surgery at the time of vaginal hysterectomy was an acknowledged challenge, previous research demonstrated that salpingectomy was feasible for the majority of women undergoing vaginal hysterectomy, as 81% of cases were reported to have successfully undergone this procedure in one publication (13). Meanwhile, the slight increase in BSO was inconsistent with the findings reported by Lai et al. (14), which presented a decreasing trend in this surgery*.* As for the number of hysterectomies with/without adnexal surgeries in each surgical method, a significant reduction in the number of AH by −17.89% accompanied by an increase in the number of TLH by 36.56% over the study period suggests a positive change in our hospital, which was consistent with the Polish study (15). There was also a sharp downward trend in the total number of hysterectomies between 2019 and 2020. There are likely several patients whose hysterectomy is being postponed due to the coronavirus (COVID-19) pandemic (16).

Patient characteristics data in our study demonstrated that the most frequent indication for hysterectomy was leiomyoma, followed by endometrial lesions, and these results were consistent with what was observed in Finland between 2001 and 2018 (10). Another important finding was that the majority of hysterectomy indications were endometrial lesions in the 25–45 age group, leiomyoma in the 45–65 age group, and uterine prolapse in the >65 age group. Uterine prolapse can affect women of any age, but it more commonly occurs in older women and increases with age, reaching a peak of 5% in women aged 60–69 (17). Furthermore, in the current research, patients were more likely to have a hysterectomy if their number of pregnancies had increased, and based on the different surgical approaches, the majority of the age criteria for hysterectomy were between 50 and 65 in AH, 45 and 50 in TLH, and >65 in VH. Leiomyoma in AH and TLH, and uterine prolapse in VH, were observed to be the most significant indications for different surgical approaches. A retrospective study including 172 women aged 45–85 who had VH for uterine prolapse found that 78% of women also underwent BSO (18), which was consistent with our findings.

In a comprehensive analysis of different surgical approaches for hysterectomy, we found that TLH was the main surgical approach for BS in this study. At the same time, the results showed that in the three surgical approaches of AH, TLH, and VH, the operative bleeding, duration of surgery, and length of hospital stay of patients undergoing TLH combined with BS and BSO were significantly lower than those of the other two groups. This is the study's primary finding. However, TLH is more widely used, probably because laparoscopic assistance allows for a clearer view of the pelvic structures (19). Comparative studies with benign indications for VH and AH have also demonstrated a better and faster improvement in the short-term quality of life after laparoscopic surgery (20).

However, our study has limitations. Observing the change in the surgical approach to hysterectomy and the type of adnexal surgery over a longer period of time with a larger sample size can make the research better. In addition, the single data center in our hospital is limited to AH, TLH, and VH patients with/without BS and BSO, restricting the generalizability of the findings. Therefore, multi-centered studies with larger sample sizes are required.

## Conclusion

From 2015 to 2021, the number of patients with hysterectomies in our hospital increased significantly, as did the incidence of THL, especially when combined with BS. TLH can reduce intraoperative blood loss and hospitalization in the majority of patients and has become the most important surgical approach for hysterectomy of benign gynecological diseases in our hospital. Therefore, emphasizing TLH surgical training is more beneficial for BS patients.

## Data Availability

The original contributions presented in the study are included in the article/Supplementary Material, further inquiries can be directed to the corresponding authors.
